# Psoas Abscess Secondary to Migrated Biliary Stent: A Rare Complication of Endoscopic Biliary Drainage

**DOI:** 10.7759/cureus.96495

**Published:** 2025-11-10

**Authors:** Sally Grice, Xavier Frisch, Sukhpal Singh

**Affiliations:** 1 General Surgery, Frimley Park Hospital, Frimley, GBR; 2 Anaesthesia, Frimley Park Hospital, Frimley, GBR

**Keywords:** biliary stent migration, duodenal perforation, endoscopic removal, iliopsoas abscess, percutaneous drainage

## Abstract

Endoscopic biliary stenting is a common and generally safe intervention for biliary obstruction. Stent migration is an uncommon complication, and extraintestinal migration resulting in abscess formation is exceptionally rare.

We describe the case of a 75-year-old woman with a past medical history of childhood polio, nummular eczema (on methotrexate) and hypertension, who developed a left iliopsoas abscess seven months after endoscopic biliary stent insertion for choledocholithiasis. Imaging revealed that the stent had migrated through the duodenal wall and inferolaterally to the left iliac bone. The abscess was drained percutaneously, and the stent was retrieved endoscopically.

This case highlights the need for timely follow-up after biliary stent insertion and complete retrieval of redundant stents where possible, and the importance of considering stent migration in patients with unexplained retroperitoneal abscesses.

## Introduction

Endoscopic retrograde cholangiopancreatography (ERCP) with biliary stent placement is widely used for the management of benign and malignant biliary obstruction. Whilst complications such as pancreatitis, bleeding, and infection are well recognised, distal stent migration occurs in approximately 4-6% of cases [1,2]. Most migrated stents pass through the gastrointestinal tract without consequence, but they can rarely perforate the bowel and migrate into adjacent structures, causing abscesses or fistulae [3].

A psoas abscess secondary to a migrated biliary stent is extremely uncommon, with very few cases reported in the literature. We report such a case to highlight this rare but important complication.

## Case presentation

A 75-year-old woman presented in March 2023 with choledocholithiasis. She underwent ERCP with sphincterotomy and the placement of two double-pigtail plastic stents in the common bile duct (CBD). Stent placement was technically challenging due to severe kyphoscoliosis secondary to poliomyelitis; consequently, the stents were left in situ for one year--longer than the usual three to six months. The stents subsequently occluded and were removed in March 2024, at which time, a single 7 Fr double-pigtail plastic stent was inserted into the CBD.

The patient re-presented in August 2024 with symptoms of cholangitis and underwent a further ERCP for another occlusion of the existing biliary stent. A stent exchange was attempted, and a 7Fr 6cm double-flanged plastic biliary stent was inserted. Retrieval of the occluded stent was again technically challenging due to severe kyphoscoliosis; the occluded stent was therefore left intragastrically for spontaneous passage through the gastrointestinal tract. The patient was discharged after a seven-day hospital admission, and a follow-up stent exchange was scheduled for one year later.

Seven months later, the patient re-presented to the Emergency Department with a one-week history of left-sided back and groin pain. On examination, she was tender over her left lower back. There was no palpable abdominal mass. Laboratory findings showed a leukocytosis, elevated inflammatory markers and deranged liver function, as displayed in Table 1.

**Table 1:** Relevant laboratory findings

Test	Result	Normal Range
White cell count	15.3	4-10 x10^9^/L
C-reactive protein	154	0-10 mg/L
Alkaline phosphotase	800	30-130 IU/L
Bilirubin	6	5-21 umol/L

The patient had no underlying risk factors for duodenal perforation, such as jejunal diverticula or steroid use. She was taking methotrexate for nummular eczema. Methotrexate is an immunomodulator and therefore has the potential to cause mucosal fragility and impaired healing, and a subsequent delay in healing or sealing of any small erosions or microperforations. However, the literature demonstrates no established link between methotrexate use and duodenal perforation from biliary stents [4].

Investigations 

**Figure 1:**
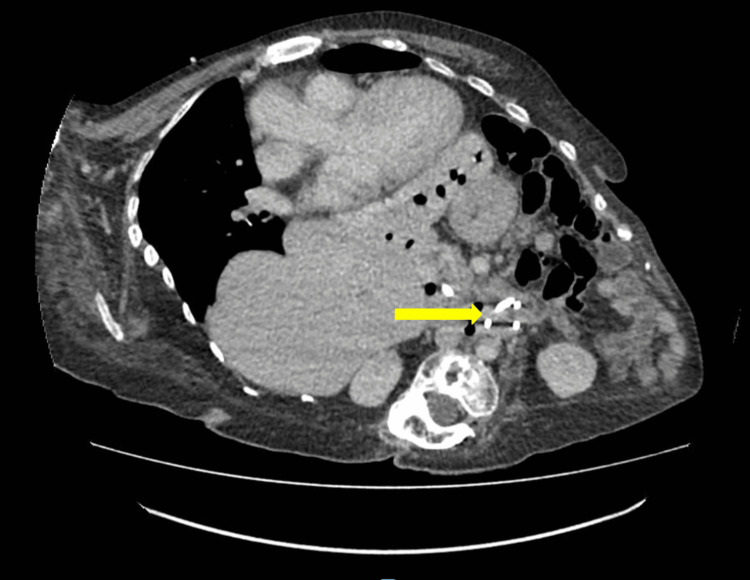
Contrast CT showing the second pigtail stent in the duodenum (yellow arrow)

The contrast-enhanced computed tomography (CT) scan of the abdomen and pelvis demonstrated a correctly placed stent in the CBD. A second stent was identified, with the proximal pigtail appearing to reside within the duodenum (Figure 1).

**Figure 2:**
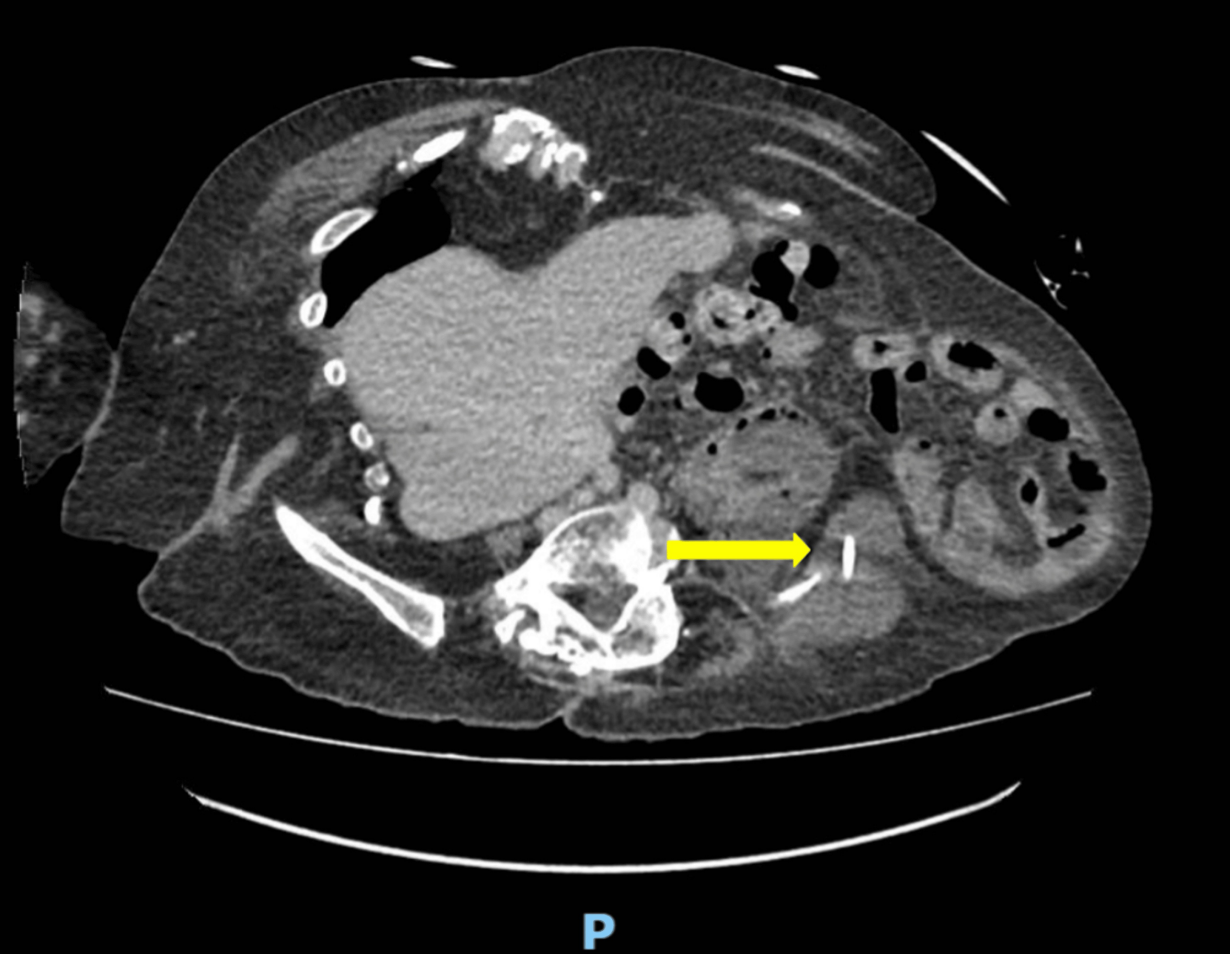
Contrast CT showing the second stent traversing the duodenal wall and extending inferolaterally (yellow arrow)

The pigtail stent then appeared to traverse the duodenal wall and extend adjacent to the left iliac bone and superior to the gluteal muscles (Figure 2). An associated 5.4cm collection was also noted.

**Figure 3:**
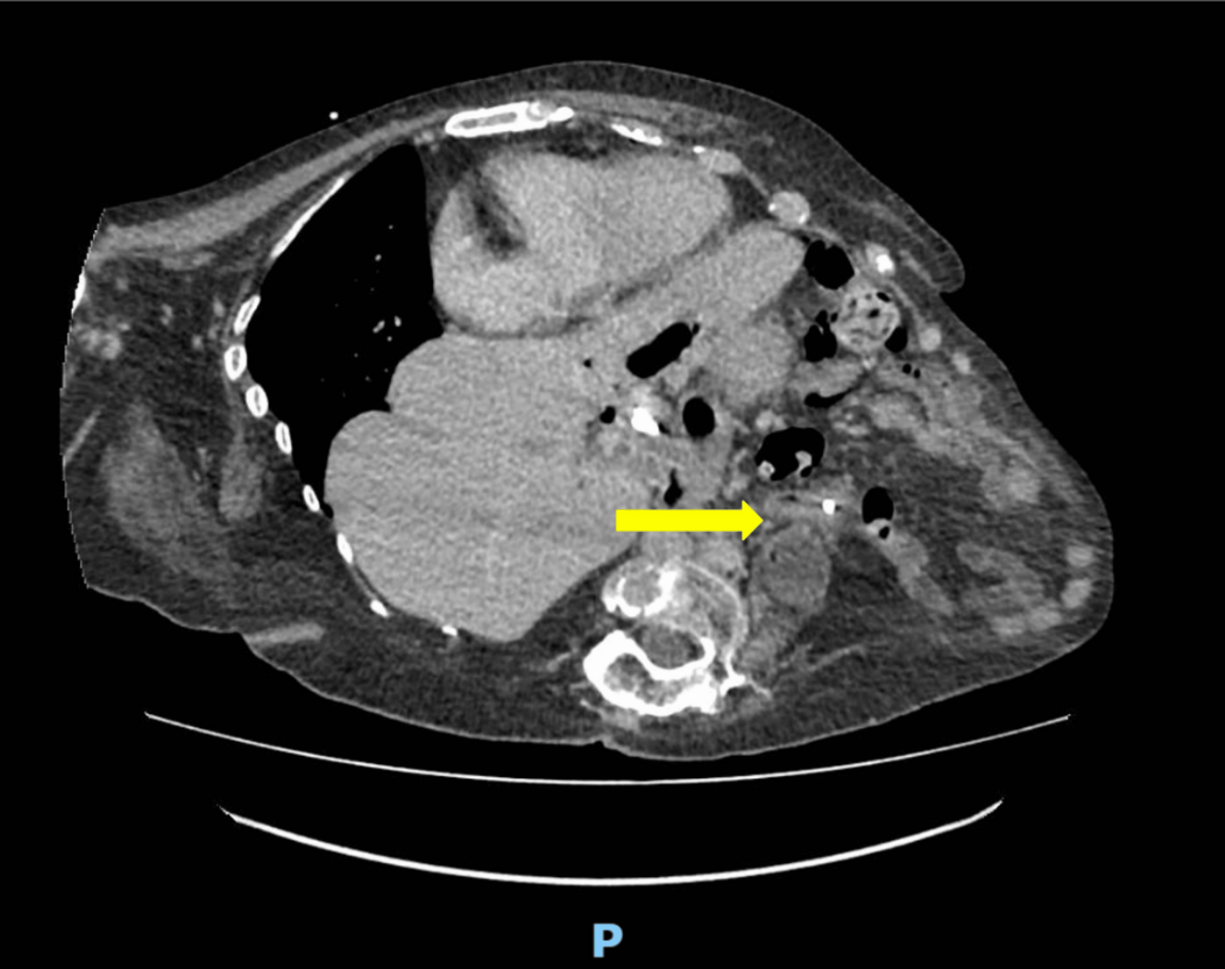
Large, left-sided iliopsoas abscess (yellow arrow) The proximal end is in close proximity to the exit site of the non-luminal stent.

Additionally, there was a large, left-sided, gas-containing iliopsoas abscess, likely secondary to the perforated duodenum (Figure 3). These findings were consistent with a migrated biliary stent that had perforated the duodenum.

Management

**Figure 4:**
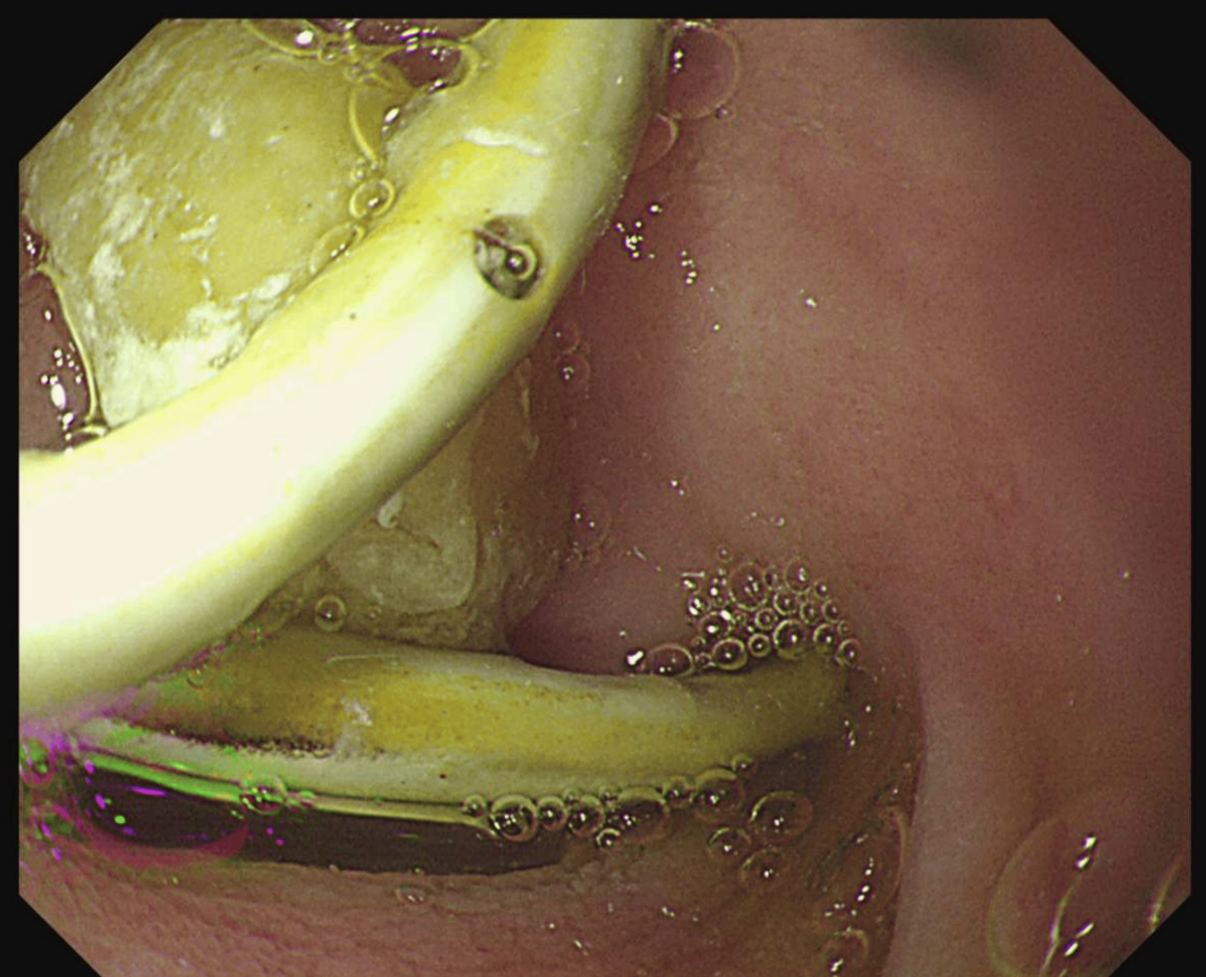
OGD image showing the biliary stent perforating the wall of the duodenum OGD: oesophago-gastro-duodenoscopy

The iliopsoas abscess was drained percutaneously under ultrasound guidance. A total of 120 ml of purulent material was aspirated, which cultured *Escherichia coli*, and broad-spectrum intravenous antibiotics were commenced. An oesophago-gastro-duodenoscopy (OGD) demonstrated the distal end of the plastic stent protruding through the duodenal wall (Figure 4).

**Figure 5:**
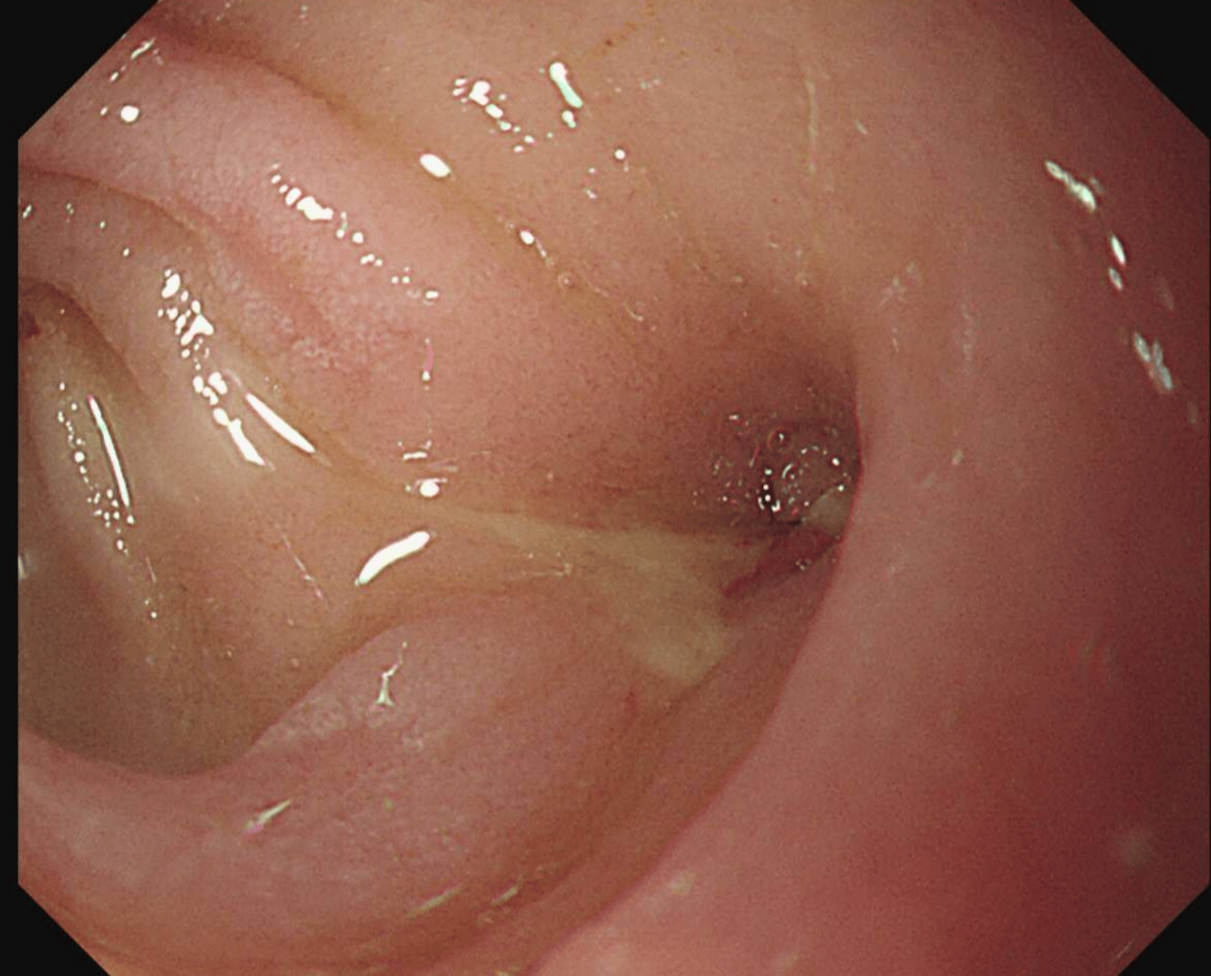
OGD image showing a small defect in the duodenal wall following endoscopic retrieval of the biliary stent OGD: oesophago-gastro-duodenoscopy

The stent was successfully retrieved endoscopically. The duodenal defect was small and managed conservatively with bowel rest and antibiotics (Figure 5).

The patient made a gradual recovery, with several repeat CT scans performed to monitor the resolution of the collection. Imaging revealed a new collection superior and posterior to the left iliac wing, which communicated with the iliopsoas abscess. A further ultrasound-guided drain was inserted into this collection, after which the patient showed clinical improvement and was discharged with a four-week course of home-administered intravenous antibiotics.

## Discussion

Migration of biliary stents is a recognised but relatively uncommon complication following ERCP, with reported rates varying depending on stent type, size, and dwell time [1]. Distal migration typically occurs into the duodenum, where the stent usually passes spontaneously without clinical consequence. However, in a small proportion of cases, the stent may become impacted or erode through the bowel wall, leading to perforation or abscess formation. Intestinal perforation secondary to stent migration is rare, occurring in fewer than 1% of reported cases [2,5]. Areas of relative fixation, such as the duodenum, ileocaecal region, and sigmoid colon, are particularly susceptible to pressure necrosis and subsequent perforation.

Once the stent breaches the bowel wall, it can migrate into adjacent anatomical compartments and serve as a nidus for infection. Retroperitoneal extension may result in the development of psoas or pararenal abscesses, which can present with non-specific symptoms, such as fever, flank pain, or difficulty mobilising the hip, often delaying diagnosis. Cross-sectional imaging, particularly contrast-enhanced CT, is invaluable for identifying both the displaced stent and associated collections. Management should be individualised based on the patient’s clinical stability, the site of perforation, and the extent of infection [3]. In haemodynamically stable patients, minimally invasive approaches, such as percutaneous drainage of abscesses combined with endoscopic retrieval of the migrated stent, are usually effective and carry lower morbidity risk compared with open surgical intervention [5]. Early recognition and prompt multidisciplinary management involving radiological and surgical teams are essential to optimise outcomes and prevent further complications.

## Conclusions

Biliary stent migration is an uncommon event but can lead to serious extraintestinal complications, including abscess formation. In patients with a history of biliary intervention, the development of a psoas abscess should raise suspicion for stent migration. Cross-sectional imaging is invaluable for diagnosis, while combined percutaneous and endoscopic management is typically effective and minimally invasive. Regular follow-up and timely removal or replacement of biliary stents are essential to prevent late complications. Whenever possible, occluded or redundant stents should be removed endoscopically rather than left for spontaneous passage, as retained plastic stents carry an increased risk of migration, obstruction, and infection. This case report reinforces the importance of adherence to stent surveillance and timely removal protocols to minimise preventable morbidity.
